# Adaptation and validation of a Korean-language version of the revised hospital survey on patient safety culture (K-HSOPSC 2.0)

**DOI:** 10.1186/s12912-020-00523-w

**Published:** 2021-01-07

**Authors:** Seung Eun Lee, V. Susan Dahinten

**Affiliations:** 1 College of Nursing, Mo-Im KIM Nursing Research Institute, Yonsei University, 50-1 Yonsei-ro, Seodaemun-gu, Seoul, 03722 South Korea; 2 School of Nursing, University of British Columbia, T201-2211 Wesbrook Mall, Vancouver, BC V6T 2B5 Canada

**Keywords:** Safety culture, Psychometric, Validity, Reliability, Korea

## Abstract

**Background:**

To date, there has been no universal and validated tool for measuring safety culture in Korea. The Hospital Survey on Patient Safety Culture (HSOPSC), version 2.0 was released by the Agency for Healthcare Research and Quality in 2019, but it had not yet been translated and assessed for use in Korea. The aim of this study was to assess the content validity and other psychometric properties of the Korean-language version of the HSOPSC 2.0.

**Methods:**

Instrument adaptation was performed using a committee-based translation, cognitive interviews, and expert panel reviews. Confirmatory factor analysis was conducted on data obtained through an online survey from 526 registered nurses who worked on medical-surgical units in three teaching hospitals in South Korea.

**Results:**

One item was dropped during the translation and adaption phase of the study as being a poor fit for the Korean healthcare context, resulting in excellent content validity. Confirmatory factor analysis supported the factorial structure of the K-HSOPSC 2.0. Correlations with an overall measure of patient safety provided further evidence of construct validity. Additionally, in comparing the results of this current study to those from U.S. research using the HSOPSC 2.0, it was found that Korean nurses assigned less positive scores to all dimensions of patient safety culture.

**Conclusion:**

Our findings provide evidence of the content validity, reliability, and construct validity of the K-HOSPSC 2.0 for measuring patient safety culture in South Korean hospitals.

Hospital administrators can use this tool to assess safety culture and identify areas for improvement to enhance patient safety and quality of care.

**Supplementary Information:**

The online version contains supplementary material available at 10.1186/s12912-020-00523-w.

## Background

Patient safety, a central principle and a determinant of healthcare quality, remains an international priority in healthcare systems. Although efforts have been made over the last two decades to improve patient safety and quality of care, a significant number of patients are still harmed while receiving health care services. One identified reason for such unsafe patient care is a weak safety culture [1]. Safety culture is an aspect of organizational culture that refers to the shared beliefs, values, and norms about safety within a healthcare organization, which impacts people’s actions and behaviors [2]. Culture influences what staff perceive as appropriate behaviors with respect to patient safety and it motivates them to engage in those behaviors [3]. Thus, assessing safety culture is an important strategy for improving patient safety and quality of healthcare [4], and safety culture is most frequently measured using a survey.

Several tools have been developed for measuring safety culture in healthcare organizations. Among them, the Hospital Survey on Patient Safety Culture (HSOPSC) developed by the Agency for Healthcare Research and Quality (AHRQ) in 2004 is the most widely used instrument internationally [4]. The 42-item HSOPSC is a self-administered questionnaire that assesses 12 dimensions of a healthcare organization’s safety culture from the perspectives of hospital staff. In 2019, the AHRQ released a revised version of the survey, the HSOPSC 2.0, comprising 32 items across 10 dimensions [5].

Healthcare organizations in Korea have made attempts to assess safety culture, but there has been no universal, validated tool for this purpose. Although translations of the original HSOPSC 1.0 or its modified versions have been commonly used to measure patient safety culture in Korea [6], the authors of those studies have failed to identify the instrument’s psychometric properties [7]. Moreover, a recent psychometric evaluation of the Korean version of the HSOPSC 1.0 [8] revealed that six of the 12 dimensions had internal consistency values below 0.70, with the lowest four Cronbach’s alphas ranging from 0.31 to 0.55. In addition, three subscales had items with poor factor loadings or cross-loadings, which may reflect shifts in meaning due to the translation or cultural differences. Notably, those items contributing to low internal consistency have been removed or revised in the newly developed HSOPSC 2.0.

The AHRQ recommends that the HSOPSC 2.0 be used instead of the original version [5] and thus, we translated the new instrument into Korean. Previous studies have emphasized the importance of careful testing of translated versions of the HSOPSC before use in order to ensure the applicability of the instrument in the target context [9]. Therefore, the aim of this study was to assess the content validity and other psychometric properties of the Korean-language version of the HSOPSC 2.0 (hereafter called the K-HSOPSC 2.0) for use in Korean hospital settings.

## Methods

### Study design

A two-phase study was conducted to translate and evaluate the K-HSOPSC 2.0. In phase 1, the instrument was translated into Korean and then each item was assessed for clarity, and cultural relevance and appropriateness (i.e., content validity). The internal consistency and construct validity of the instrument was examined in phase 2.

### Instrument

Revision of the HOSPSC 1.0 was based on feedback and recommendations from users and stakeholders, and there are notable changes in the new survey [5]. The number of safety culture items was reduced from 42 to 32, and the number of dimensions (i.e., subscales) was reduced from 12 to 10. Two dimensions, *overall perceptions of patient safety* and *teamwork across units*, with four items each, were deleted in version 2.0. Many of the remaining items were reworded, particularly those that were difficult to translate. Two pilot tests were conducted during the revision process. An initial pilot test in 44 hospitals in the United States (U.S.) in 2017 led to further modifications and a second pilot test in 25 hospitals in 2019; this version became the HSOPSC 2.0 and was released to the public in 2019 [10]. Cronbach’s alphas for the 10 subscales of the HSOPSC 2.0 ranged from 0.67 to 0.89 in the U.S. study [11]. Table 1 lists the 10 safety culture dimensions and related items.

**Table 1:** Dimensions and Items of the Hospital Survey on Patient Safety Culture version 2.0

Dimension		Item	
1	Teamwork	A1	In this unit, we work together as an effective team
		A8	During busy times, staff in this unit help each other
		A9r	There is a problem with disrespectful behavior by those working in this unit
2	Staffing and Work Pace	A2	In this unit, we have enough staff to handle the workload
		A3r	Staff in this unit work longer hours than is best for patient care
		A5r^a^	This unit relies too much on temporary, float, or PRN staff
		A11r	The work pace in this unit is so rushed that it negatively affects patient safety
3	Organizational learning – Continuous improvement	A4	This unit regularly reviews work processes to determine if changes are needed to improve patient safety
		A12	In this unit, changes to improve patient safety are evaluated to see how well they worked
		A14r	This unit lets the same patient safety problems keep happening
4	Response to Error	A6r	In this unit, staff feel like their mistakes are held against them
		A7r	When an event is reported in this unit, it feels like the person is being written up, not the problem
		A10	When staff make errors, this unit focuses on learning rather than blaming individuals
		A13r	In this unit, there is a lack of support for staff involved in patient safety errors
5	Supervisor, Manager, or Clinical Leader Support for Patient Safety	B1	My supervisor, manager, or clinical leader seriously considers staff suggestions for improving patient safety
		B2r	My supervisor, manager, or clinical leader wants us to work faster during busy times, even if it means taking shortcuts
		B3	My supervisor, manager, or clinical leader takes action to address patient safety concerns that are brought to their attention
6	Communication About Error	C1	We are informed about errors that happen in this unit
		C2	When errors happen in this unit, we discuss ways to prevent them from happening again
		C3	In this unit, we are informed about changes that are made based on event reports
7	Communication Openness	C4	In this unit, staff speak up if they see something that may negatively affect patient care
		C5	When staff in this unit see someone with more authority doing something unsafe for patients, they speak up
		C6	When staff in this unit speak up, those with more authority are open to their patient safety concerns
		C7r	In this unit, staff are afraid to ask questions when something does not seem right
8	Reporting Patient Safety Event	D1	When a mistake is caught and corrected before reaching the patient, how often is this reported?
		D2	When a mistake reaches the patient and could have harmed the patient, but did not, how often is this reported
9	Hospital Management Support for Patient Safety	F1	The actions of hospital management show that patient safety is a top priority
		F2	Hospital management provides adequate resources to improve patient safety
		F3r	Hospital management seems interested in patient safety only after an adverse event happens
10	Handoffs and Information Exchange	F4r	When transferring patients from one unit to another, important information is often left out
		F5r	During shift changes, important patient care information is often left out
		F6	During shift changes, there is adequate time to exchange all key patient care information
	Number of Events Reported^b^	D3	In the past 12 months, how many patient safety events have you reported?
	Patient Safety Rating^b^	E1	How would you rate your unit/work area on patient safety?

*r* negatively worded item

^a^ A5r was removed from the final Korean version of the survey as it does not fit the Korean context. ^b^ single item measure

The 32 safety culture items in the HSOPSC 2.0 questionnaire are measured on 5-point response scales in terms of agreement (strongly disagree to strongly agree) or frequency (never to always), as well as an option for “does not apply or do not know”. There are also two single items that ask respondents (1) to provide an overall rating of patient safety for their unit (i.e., *a patient safety grade*) using a 5-point response scale (poor to excellent), and (2) how many patient safety events they have reported.

### Translation and content validity of the HSOPSC 2.0

After receiving permission from the AHRQ, the translation process began. We used a committee-based translation approach, which helps to achieve cultural consensus when the languages are linguistically quite different [12]. The translation committee members consisted of three bilingual Korean nursing professors and one bilingual Korean hospital nurse; all four had worked as health professionals in both the U.S. and Korea and thus, were familiar with both cultures. First, the committee members independently translated the HOSPSC 2.0. After individual translation, the committee reviewed the four translated versions, discussed ambiguities and discrepancies, and adjudicated the final version of the K-HSOPSC 2.0.

Semi-structured, face-to-face cognitive interviews were then conducted with 10 direct care nurses who worked on medical/surgical units to evaluate their understanding, and the clarity of the translated items, response options, and survey instructions [13]. For items deemed unclear, participants were asked to provide suggestions to improve clarity. Minor modifications were made based on the feedback provided during the interviews.

The questionnaire was also reviewed by an expert panel comprised of 10 patient safety experts in academic and clinical settings. The experts individually rated the cultural relevance and appropriateness of each translated item using a 4-point scale ranging from 1 (not relevant) to 4 (highly relevant). The results of the expert panel reviews were used to finalize the content of the K-HOSPSC 2.0 and evaluate its content validity. A content validity index (CVI) was calculated for each item and the total scale. Each item CVI (I-CVI) score was calculated using the percentage of experts who rated the item as 3 or 4, and the scale-CVI (S-CVI) was calculated by computing the mean of the I-CVI scores. I-CVI scores above 0.80 are considered acceptable, and an S-CVI score above 0.90 is considered excellent [14]. The expert panel agreed that item A5 does not reflect staffing models in Korean hospitals as the use of temporary, float, or PRN staff is highly uncommon, and thus, the item was deleted from the survey. It is notable that the *staffing* subscale in the Korean version of the HOSPSC 1.0 was also found to be problematic, having a Cronbach’s alpha of 0.31, and it was strongly recommended that the subscale be revised [8]. Excluding the A5 item on the K-HSOSPC 2.0 yielded I-CVI scores ranging from 0.80 to 1.00 and an S-CVI of 0.96, indicating excellent content validity.

### Setting, sample, and data collection

For phase 2, we used convenience sampling to recruit direct care nurses from three hospitals in South Korea. The hospitals were located in one of two cities, and each had a minimum total bed capacity of 1000. Only registered nurses who had at least 6 months nursing experience and were working on medical/surgical units were able to participate in the study.

Due to the COVID-19 outbreak, an online survey was carried out to avoid personal contact during data collection. Recruitment notices with a link to the online questionnaire were distributed to 731 nurses by the hospitals, on behalf of the research team. Study participants were informed about the study aims and methods, the voluntary nature of participation, and confidentiality of the responses, and were offered a gift certificate (equivalent to US$9) as honorarium for participation. In addition to the K-HSOPSC 2.0 safety culture items, the survey included seven demographic questions that asked sex, age, years of nursing experience, education level, hospital tenure, unit tenure, and employment type (permanent full-time or temporary full-time). A total of 526 nurses completed the online survey between May and June 2020, for a 72% response rate.

Determining the sample size requirements for confirmatory factor analysis (CFA) remains a challenge as it is impacted by the total number of factors and indicators, as well as the size of factor loadings [15]. Recommendations for sample size have ranged from ratios of 5 to 20 cases per item [16], and from 50 participants for simple CFA models (Furr, 2018) to 500 cases [17]. Thus, our sample size of 526 (with about 16.5 cases per indicator) was considered suitable for gaining a stable factor solution for the 31 item K-HOSPSC 2.0. This study was approved by the respective Institutional Review Board of the relevant university (#Y-2020-0013) and was conducted in accordance with the Code of Ethics of the World Medical Association (Declaration of Helsinki).

### Data analysis

All negatively worded items were reverse-coded for statistical analyses. Descriptive statistics were computed for participant characteristics and each K-HOSPSC 2.0 subscale. To compare the findings between the current survey and the original U.S. survey, the percentage of positive responses for each subscale were calculated as recommended by the tool developers [18]. For this, we calculated the mean percentage of respondents who answered “strongly agree” or “agree”, or “always” or “most of the time” for positively worded items, and answered “strongly disagree” or “disagree”, or “never” or “rarely” for negative worded items. Cronbach’s alphas were calculated to assess the internal consistency of each subscale, with 0.7 generally considered the minimum criterion for acceptable reliability [19].

The factor structure of the K-HOSPSC 2.0 was examined through CFA using maximum likelihood estimation. As recommended by Kline [20], we evaluated the following goodness-of-fit indices of the measurement model: root mean square error of approximation (RMSEA), standardized root mean residual (SRMR), and comparative fit index to (CFI). An RMSEA value < 0.06 and an SRMR value < 0.08 are considered a good fit [21]. A CFI value ≥0.9 indicates a good fit and a value ≥0.8 indicates an acceptable fit [22]. Pearson correlation analysis was used to examine inter-correlations among the 10 patient safety subscales. Correlations greater than 0.7 would indicate that the subscales were measuring the same concept and those subscales could be combined and/or some items could be removed [23].

To provide further evidence of construct validity, Spearman rho correlation coefficients were used to examine the relationships between the 10 K-HSOPSC 2.0 subscales and the single item that measured *patient safety grade* [23, 24]. Statistical analyses were performed using the STATA version 16.1 with a significance level of *p* < .05.

## Results

### Participants’ characteristics

As shown in Table 2, 98% of the 526 participants were female, with a mean age of 31 years (*SD* = 11). The majority (89%) had a baccalaureate or higher degree in nursing. Except for three respondents, all were permanent, full-time employees. On average, participants had 7.5 years of nursing experience (*SD* = 6.5), and had worked in the hospital 7.1 years (*SD* = 6.5), and on their current unit 4.4 years (*SD* = 3.9).

**Table 2:** Demographic Characteristics of Study Participants (*N* = 526)

Characteristic	Number	Percent	*M*	*SD*
Gender				
Male	9	1.71		
Female	517	98.29		
Education				
Associate degree	25	4.75		
Bachelor’s degree	442	84.03		
Master’s degree and above	59	11.22		
Employment type				
Permanent, full-time	523	99.43		
Temporary, full-time	3	0.57		
Age in years			31.20	11.28
Years in nursing			7.53	6.52
Years in current hospital			7.07	6.46
Years in current unit			4.39	3.88

### Descriptive statistics for nurse-perceived patient safety culture

Table 3 presents the descriptive statistics for the K-HSOPSC 2.0. The two lowest mean scores were 2.39 for *staffing and work pace* and 2.72 for *response to error* (indicating disagreement or a negative perception of these issues). The two highest mean scores were 3.71 for *supervisor, manager or clinical leader support of patient safety* and 3.59 for *communication about error* (indicating agreement or a positive perception of these issues). The percentage of positive responses is also reported on Table 3. Compared to the results from the original U.S. study [10], Korean nurses reported much lower safety culture scores for each subscale. Table 3 also shows that the Cronbach’s alphas were above 0.70 for all subscales except *staffing and work pace* (α = 0.61).

**Table 3:** Descriptive Statistics for the K-HSOPSC 2.0

Subscale (number of items)	*M (SD)*	Cronbach’s α		Percentage of Positive Responses	
		This study	HSOPSC 2.0	This study	HSOPSC 2.0
Teamwork (3)	3.54 (0.61)	0.77	0.76	62	81
Staffing and Work Pace (3)	2.39 (0.61)	0.61	0.67	13	56
Organizational Learning – Continuous Improvement (3)	3.42 (0.57)	0.71	0.76	54	72
Response to Error (4)	2.72 (0.65)	0.72	0.83	22	61
Supervisor, Manager, or Clinical Leader Support for Patient Safety (3)	3.71 (0.61)	0.75	0.77	69	81
Communication about Error (3)	3.59 (0.67)	0.83	0.89	50	68
Communication Openness (4)	3.24 (0.58)	0.73	0.83	38	76
Reporting Patient Safety Events (2)	3.30 (0.74)	0.73	0.75	40	74
Hospital Management Support for Patient Safety (3)	2.94 (0.72)	0.72	0.77	31	68
Handoffs and Information Exchange (3)	3.39 (0.63)	0.72	0.72	53	58

*HSOPSC* Hospital Survey on Patient Safety Culture

### Confirmatory factor analysis results

All items within each factor had acceptable factor loadings above 0.4, ranging from 0.46 to 0.86 (Supplemental Figure) [25]. As shown in Table 4, the fit indices indicated that the 10-factor model provided an acceptable fit to the data: χ2/df ratio = 2.51 (χ2 = 978.37 df = 389, *p* < 0.001), RMSEA = 0.05, SRMR = 0.06, and CFI = 0.89. Confirmation of the factorial structure of an instrument provides one type of evidence that is important for establishing an instrument’s construct validity [16].

**Table 4:** Fit indices for the K-HSOPSC 2.0

Fit indices	
CFI (Comparative Fit Index)	0.89
RMSEA (Root Mean Squared Error of Approximation)	0.05 (Confidence interval: 0.05–0.06)
SRMR (Standardized Root Mean Residual)	0.06
χ^2^/df	2.51 < 5

The Pearson correlation coefficients among the 10 subscales ranged from 0.19 to 0.45 (*p* < 0.05), indicating sufficient independence among the subscales. The strongest correlation was between *supervisor, manager, or clinical leader support for patient safety* and *communication openness* (*r* = 0.45) and the lowest correlation was for *staffing and work pace* and *supervisor, manager, or clinical leader support for patient safety* (*r* = 0.19) as well as *communication openness* (*r* = 0.19). No exceptionally high correlations were noted. Also, the 10 subscales were positively correlated with patient safety grade, with correlations ranging from 0.19 to 0.37, providing further evidence of the construct validity of the instrument.

## Discussion

In this study, we translated and adapted the HSOPSC 2.0 to the Korean healthcare context, and examined the psychometric properties of the instrument. In general, study findings showed good internal consistency, content validity, and construct validity, indicating that the K-HSOPSC 2.0 can be used to measure staff nurses’ perceptions of patient safety culture in Korean hospitals.

A rigorous process was followed for instrument translation and assessment of content validity, using a committee-based translation method, cognitive interviews, and expert panel reviews. We deleted one item (A5), “this unit relies too much on temporary, float, or PRN staff,” from *Staffing and work pace* subscale based on feedback from the interviewees and expert panel. Thus, the subscale in the K-HSOPSC 2.0 has only three items rather than four as shown in the U.S. version [5]. In contrast to U.S. hospitals, in Korea, the use of float staff is uncommon, and nurses (including temporary staff) have fixed-term contracts (e.g., one year of full-time work). Thus, this item may seem confusing or be irrelevant in Korean healthcare systems. In the current study, the internal consistency coefficient for the 3-item *staffing and work pace* subscale was 0.61; the alpha value for the subscale was 0.67 in the U.S. version. Although the subscale did not reach the 0.70 threshold recommended by Nunnally and Bernstein [19], other more recent resources have considered Cronbach’s alpha coefficients greater than 0.6 to be acceptable [23, 26]. Thus, our findings support deletion of item A5 from the HSOPSC 2.0 for use in Korean hospitals. Cronbach’s alphas for the other nine subscales ranged from 0.71 to 0.83, demonstrating good internal consistency of each subscale.

The results of the CFA for the 31 items of the translated instrument supported the 10-factor structure of the HOSPSC 2.0. Each item contributed to its expected subscale, providing evidence of construct validity. In addition, as hypothesized, all subscales were associated with *patient safety grade*, which provides further evidence of the construct validity of the K-HSOPSC 2.0 [16].

In comparing our results to those from the U.S. study [10], we found that Korean nurses assigned less positive scores to all dimensions of patient safety culture. Notably, *staffing and work pace* received the lowest mean score with only 13% of respondents in this study reporting that their work unit had adequate staffing, compared with 56% in the U.S. These findings are similar to the findings from previous Korean research. Nurse staffing has long been a problem in the Korean healthcare systems. Cho and colleagues [27] found that the average nurse-patient ratio was 1:16 in general hospitals and approximately 1:17 in general units with 50 patient beds [28], a significantly higher patient load than the minimum nurse-patient ratios of 1:4 and 1:5 that were mandated in 2004 for general medical-surgical units in California, U.S. [29]. South Korea does have a national standard of 2.5 registered nurses per daily patient census, but Cho et al. [27] found that only 63% of general hospitals were adhering to this standard. *Response to error* received the second lowest rating, consistent with other Korean studies [30, 31]. Only 22% of the participants in this study reported that they agreed or strongly agreed that their work culture was not punitive, compared with 61% in the U.S. study.

The low ratings for *staffing and work pace* and *response to error* are problematic as adequate staffing and a nonpunitive response to errors are important for improving patient safety and quality of care [4, 32]. Thus, nurse managers should advocate for safe staffing levels, and hospital administrators should invest in the nurse staffing levels that are necessary for safe patient care. Also, the Korean government should revisit the national standards for minimum nurse staffing levels and monitor hospital compliance with those standards [27]. To create nonpunitive work environments, hospital administrators and nurse managers should demonstrate openness to change, foster open communication, and establish safe environments where nurses can freely discuss patient safety–related issues and errors, which will, in turn, allow the organization to learn from those errors and improve the quality and safety of patient care [33].

The findings of this study support the use of the K-HOSPSC 2.0 for investigating patient safety culture in Korean hospitals. This tool can assist hospital administrators and nurse managers to identify areas for improvement to enhance patient safety and quality of care. Using this tool, hospital administrators and nursing managers will be able to benchmark their results against that of other units, hospitals, and countries; to date, such comparisons have been limited due to the lack of reliable and valid instrument for measuring patient safety culture in Korea [6]. The preliminary findings from our study also suggest that nurse managers should advocate for increased nurse staffing levels, and attend to the manner in which they respond, or are perceived to respond, to patient safety errors.

### Limitations

Although our study provides evidence of the reliability and construct validity of the K-HSOPSC 2.0 for measuring patient safety culture in Korean hospitals, some limitations should be noted. First, the respondents in this study worked in three tertiary general hospitals, all of which are affiliated with medical schools. In addition, all respondents were direct care staff nurses working in medical/surgical units, and the majority were female. Thus, the results might not represent all Korean nurses and cannot be generalized to other healthcare professionals. Therefore, the psychometric properties of this instrument should be examined in a broader validation context in future research. Second, stability over time was not assessed as a second administration of the instrument was not considered feasible by the hospital managers at this time, in the midst of the coronavirus pandemic. Third, to our best knowledge, there have not yet been any published papers that examine the psychometric properties of the HSOPSC 2.0, and thus we were not able to compare our results to other published studies of the instrument. Finally, we acknowledge that the use of a committee-based approach to cross-cultural translation remains somewhat controversial, although its use has been supported by other research literature [34, 35].

## Conclusions

To our knowledge, this is the first study to investigate the psychometric properties of the HSOPSC 2.0 in the Korean healthcare context. Our findings provide preliminary evidence of the internal consistency, content validity, and construct validity of the translated and adapted instrument, based on data from direct care nurses working on medical-surgical units in Korean hospitals. In particular, our CFA results support the use of the 31-item, 10-factor K-HSOPSC 2.0. However, more research is needed to investigate its psychometric properties within a broader validation context.

## Supplementary Information

**Additional file 1: Supplemental Figure.** Standardized parameter estimates for the factor structure of the K-HSOPSC 2.0.

## Data Availability

The datasets generated and/or analysed during the current study are not publicly available due to them containing information that could compromise research participant consent but are available from the corresponding author on reasonable request.

## Abbreviation

HSOPSC: Hospital Survey on Patient Safety Culture
