# Supplementary figures and images for: Adaptation and validation of a Korean-language version of the revised hospital survey on patient safety culture (K-HSOPSC 2.0)
Source: BMC Nurs. 2021 Jan 7;20:12. doi: 10.1186/s12912-020-00523-w (PMC7790030; doi:10.1186/s12912-020-00523-w)

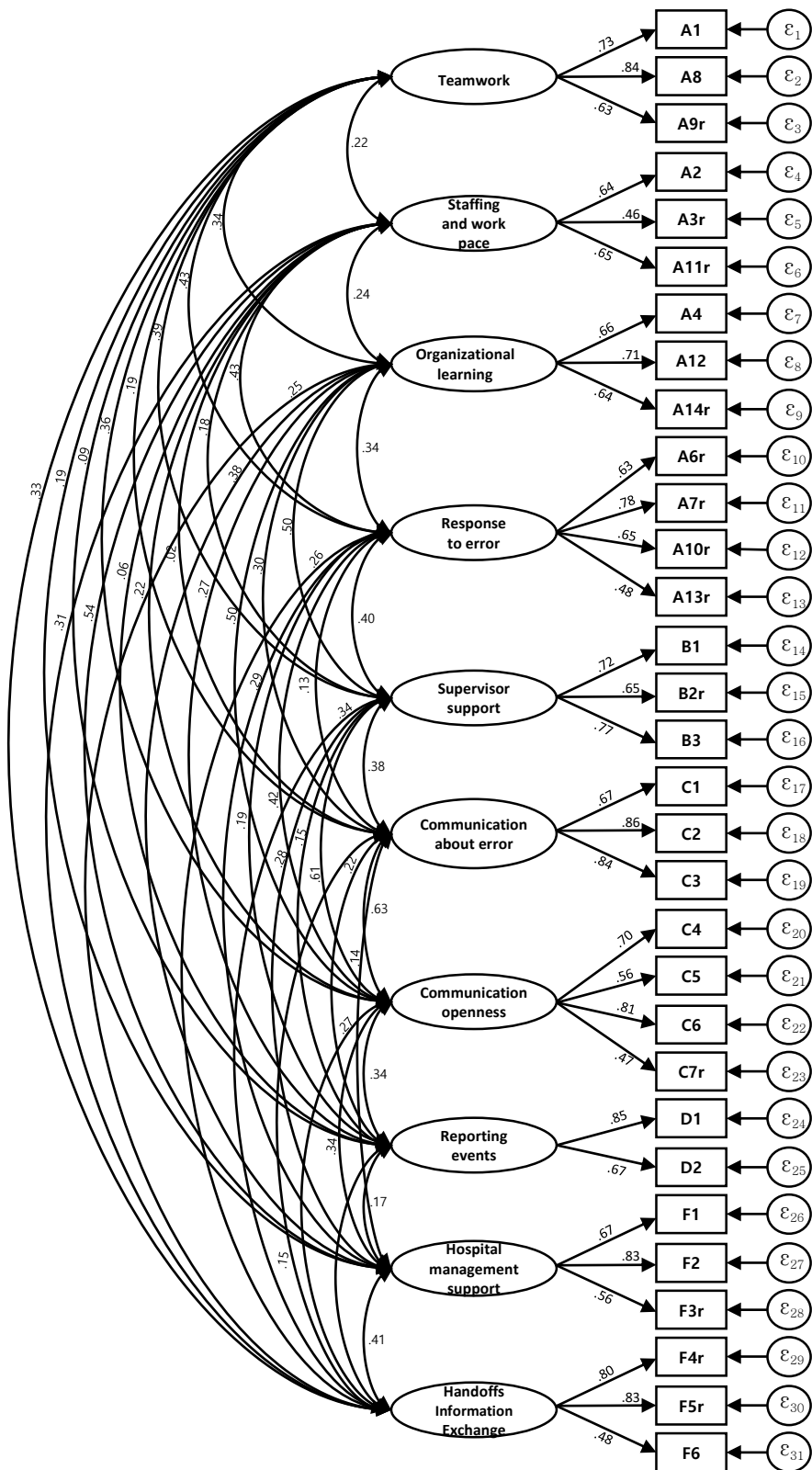

Supplement: Supplementary file 1 — Additional file 1: Supplemental Figure. Standardized parameter estimates for the factor structure of the K-HSOPSC 2.0. [file 12912_2020_523_MOESM1_ESM.pdf]
